# Editorials

**Published:** 1894-09

**Authors:** 


### HOMŒOPATHIC PHYSICIAN,

A MONTHLY JOURNAL OF

HOMŒOPATHIC MATERIA MEDICA AND CLINICAL MEDICINE.

"If our school ever gives up the strict inductive method of Hahnemann, we are lost, and deserve only to be mentioned as a caricature in the history of medicine."—CONSTANTINE HERING.

Vol. XIV. SEPTEMBER, 1894. No. 9.

### EDITORIALS.

THE USE AND THE ABUSE OF ACONITE.—No remedy in the homœopathic materia medica is so universally used alike by the doctors and the laity as Aconite. Whenever a patient has fever, Aconite is at once prescribed. Fever is regarded by the profession as a symptom that takes precedence of all others, and must be "vigorously attacked." So Aconite is prescribed in frequent doses of the lowest potencies to "break the fever." It matters not what the other symptoms may be, Aconite is given without regard to them.

Careless practitioners who are too indolent to study, or too busy to search out the remedy in the materia medica, give Aconite without taking any time to consider the case.

Whenever the doctor does not know the indicated remedy he gives Aconite, and justifies himself by reflecting that Aconite is always indicated in fever.

Most of the books on practice lay particular emphasis upon Aconite whenever there is fever.

Nearly all the books of domestic practice advise its "early exhibition" whenever there is fever. So the principal idea in the mind of the household matron when her offspring is taken suddenly sick in the middle of the night is the fever. This is the principal symptom to be combatted, and she at once resorts to the Aconite bottle, from which she administers frequent doses to control the fever "until the doctor comes."

Thus has arisen the proverb: "Aconite is the homœopathic lancet."

Now Aconite is not the most frequently indicated remedy. Many other remedies have fever as strongly marked as has Aconite.

Moreover, Aconite has two or three characteristic symptoms which are not invariably found in those who have fever.

One of these symptoms is *fear of death. The patient predicts the day he is to die.*

Another symptom is the *restlessness, thirst, and anxiety.* If the patient be a child it will hardly show the first-mentioned symptom—fear of death. But the anxiety, the thirst, and the restlessness appear as soon as night falls.

*Aggravation at night* is then another characteristic of Aconite.

The restlessness is peculiar. As described by the late Dr. Lippe, it is a rolling and tumbling about the bed "just like a kitten." The restlessness of Apis is of the same character. The Arsenic patient, on the other hand, is possessed with a restlessness which drives him out of bed, from one bed to another, or from one chair to another, or from one room to another. The Aconite patient remains in bed, but tumbles about the bed and rolls over and over.

The Aconite patient has a red face—a bright red face, whilst Belladonna has a purple-red face and a restlessness similar to Aconite.

Now when you have the foregoing group of symptoms: high fever with bright red face, intense restlessness as previously described as soon as night comes on, great thirst, great anxiety, and fear of death, you have the great characteristics of Aconite which will make it the true simillimum for any case of acute illness that may come under the physician's notice.

This is the view of Aconite held by the late Dr. Lippe, constantly insisted upon by him, in many lectures which he delivered in the Homœopathic Medical College of Pennsylvania—

the predecessor of the present Hahnemann College of Philadelphia—in many discussions in the societies to which he belonged, and in private conversation.

The editor of this journal was intimately associated with Dr. Lippe for twenty years; was his confidant, assistant, and medical attendant. He has frequently heard him speak strongly of the "abuse of Aconite" by the majority of the medical profession, and declare that those who practiced Homœopathy loosely gave it more frequently than any other remedy, while those who practiced closely according to the most refined differential indications rarely used this remedy because the above-mentioned group of symptoms was rarely observed.

---

The Delinquency of the Subscribers to The Homœopathic Physician in the important duty of paying their annual dues to the journal is again causing the editor considerable annoyance. It is not pleasant to be constantly writing dunning letters to those who are in debt to the journal. Nor is it profitable to the readers to have valuable space taken up in its pages with appeals for payment. Certainly the material given is worthy the time taken to read and study it, else our patrons would not continue their subscriptions. Neither would they so carefully hoard up the numbers and put them into good bindings.

Therefore it would seem that they would be only too happy to show their appreciation of what is prepared for them by a cheerful and prompt remittance. We now once more appeal to those who owe for one or more years' subscriptions that they will, upon reading this notice, forward the amounts due without further loss of time, and without further need of writing personal letters.

---

Wells on Intermittent Fever comes to an end with this number. It will shortly be bound and for sale in a separate volume. In its place we shall publish in our next number the third chapter of Dr. Lee's long-delayed *Repertory of Char-*

*acteristics.* This chapter will be entitled "Vertigo," and will be devoted exclusively to the varieties and conditions of that symptom. This relegating of vertigo to a special chapter is somewhat of an innovation upon the usual arrangement of repertories that are divided into chapters, and may cause criticism. It is, however, deemed best for the purpose in view, the selection of the simillimum. The type is now being set and the proof scanned for the next issue.

